# Radiographic medial posterior tibial slope ≥16° predicts multiple revisions after anterior cruciate ligament reconstruction

**DOI:** 10.1002/jeo2.70624

**Published:** 2026-01-19

**Authors:** Mahmut Enes Kayaalp, Jumpei Inoue, Efstathios Konstantinou, Hamit Çağlayan Kahraman, Tunay Erden, Volker Musahl

**Affiliations:** ^1^ Department of Orthopaedic Surgery, UPMC Freddie Fu Sports Medicine Center University of Pittsburgh Pittsburgh Pennsylvania USA; ^2^ Department of Orthopaedics and Traumatology University of Health Sciences, Istanbul Fatih Sultan Mehmet Training and Research Hospital Istanbul Türkiye; ^3^ Faculty of Health Sciences Brandenburg, Brandenburg Medical School Theodor Fontane Brandenburg an der Havel Germany; ^4^ Department of Orthopaedic Surgery Nagoya Tokushukai General Hospital Kasugai Aichi Japan; ^5^ Department of Orthopaedics and Traumatology University of Thessaly, University Hospital of Larissa Larissa Greece; ^6^ Department of Orthopaedics and Traumatology Acıbadem Fulya Hospital Sports Medicine Center Istanbul Türkiye; ^7^ Department of Orthopaedics and Traumatology Istanbul Health and Technology University Istanbul Türkiye

**Keywords:** anterior cruciate ligament reconstruction, lower extremity deformities, osteotomy, posterior tibial slope, radiography

## Abstract

**Purpose:**

An increased posterior tibial slope (PTS) has been implicated as a risk factor for anterior cruciate ligament (ACL) graft failure. This matched case–control study aimed to compare radiographic and magnetic resonance imaging (MRI)‐based PTS measurements between patients undergoing multiple revision anterior cruciate ligament reconstruction (ACLR) and those with successful primary ACLR and to identify thresholds predictive of high revision risk.

**Methods:**

In this matched case–control study, 156 patients were analysed: 78 patients undergoing multiple revision ACLR and 78 patients with successful primary ACLR. Medial PTS was measured on radiographs, while medial, lateral and PTS difference (PTS asymmetry) were measured on MRI. Group differences were assessed using independent *t* tests and *χ* ^2^ tests. Receiver operating characteristic (ROC) analysis identified optimal thresholds, and logistic regression quantified odds ratios (ORs) for multiple revisions per 1° increase in radiographic medial PTS, adjusting for body mass index (BMI), sex, side, height and weight.

**Results:**

Radiographic medial PTS was significantly higher in the multiple‐revision group (12.5 ± 3.5° vs. 11.2 ± 3.0°, *p* = 0.016). ROC analysis identified an optimal medial PTS cutoff of 13° (area under the curve = 0.58, sensitivity = 0.49, specificity = 0.65), but only a PTS ≥ 16° was significantly associated with increased multiple revision risk (OR = 3.10, 95% confidence interval [CI]: 1.14–8.40; *p* = 0.037; specificity = 0.91; positive predictive value [PPV] = 0.70). MRI‐based medial and lateral PTSs, as well as PTS asymmetry, did not differ significantly between groups. Univariate logistic regression demonstrated a 10% increase in odds per 1° increase in radiographic PTS (OR = 1.10, 95% CI: 1.00–1.22, *p* = 0.049), remaining significant after adjustment for BMI, sex, side, height and weight (adjusted OR = 1.11, 95% CI: 1.01–1.23, *p* = 0.034). Radiographic medial PTS correlated moderately with MRI‐based medial PTS (*r* = 0.49, *p* < 0.001), but not with lateral PTS (*p*: n.s.).

**Conclusion:**

Radiographic medial PTS showed the strongest differentiation between successful primary ACLR and multiple‐revision ACLR. A PTS ≥ 16° identifies patients at significantly higher risk of multiple revisions, whereas MRI‐based medial PTS, lateral PTS and PTS asymmetry provide no additional discriminatory value. Radiographic medial PTS appears practical for preoperative risk stratification, whereas MRI‐based measures do not show similar utility.

**Level of Evidence:**

Level III.

**Abbreviations**
ACL: anterior cruciate ligament
ACL‐R: anterior cruciate ligament reconstruction
ACWO: anterior closing wedge osteotomy
AUC: area under the curve
ICC: intraclass correlation
IRB: institutional review board
PTS: posterior tibial slope
ROC: receiver operating characteristic

## INTRODUCTION

Among the anatomical factors contributing to recurrent anterior cruciate ligament (ACL) reconstruction failure, an increased posterior tibial slope (PTS) has been identified as a potential risk factor for both primary and revision anterior cruciate ligament reconstruction (ACLR) failure [2, 16, 28]. Consequently, PTS‐decreasing osteotomies have recently emerged as a surgical strategy to mitigate this risk [4, 16, 17, 19]. However, considerable variability exists in the literature regarding optimal measurement techniques, threshold values and the relative importance of medial versus lateral PTSs [9, 14, 16, 26]. Reported threshold values associated with elevated risk span a broad range—typically from ≥10° up to ≥ 17°—reflecting differences in imaging modality, measurement methodology and the specific outcome assessed (e.g., primary injury, graft failure or multiple revisions) [6, 16, 24]. This heterogeneity has limited the establishment of a consistent, clinically meaningful cutoff for routine risk stratification.

Radiographic and magnetic resonance imaging (MRI) derived PTS values often differ substantially due to differences in imaging planes, landmarks and measurement methodologies. Previous studies have reported higher average PTS values on radiographs compared with MRI, but whether these modalities provide similar discriminatory value for identifying high‐risk patients remains unclear [8, 11, 12]. Furthermore, the clinical relevance of lateral PTS measurements and PTS asymmetry, that is, the difference between medial and lateral PTS, has been debated, with several studies suggesting associations with graft failure, laxity or residual laxity after ACLR, while others report no independent predictive value [13, 18, 23, 29].

The purpose of this matched case–control study was to compare radiographic and MRI‐derived medial and lateral PTS measurements between patients undergoing multiple ( ≥ 2) revision ACLR and those with successful primary ACLR. We aimed to determine which measurement modality best differentiates high‐risk patients and to identify clinically relevant thresholds associated with an increased risk of multiple revisions. We hypothesized that radiographic medial PTS would demonstrate superior predictive value compared with MRI‐derived measurements and that a radiographic medial PTS threshold would emerge as a reliable indicator of multiple‐revision risk.

## METHODS

Ethical approval was obtained prior to study start (IRB:STUDY19030196). Patients were identified within a single healthcare system over a 10‐year period, with all procedures performed by seven high‐volume sports medicine surgeons. A total of 504 patients with revision ACL reconstruction surgery were identified through the years 2013–2023, of which 94 (19%) involved multiple ( ≥ 2) revisions. Eleven patients (12%) were excluded due to lack of proper lateral radiograph of the knee, that is, ≤5 mm posterior or distal femoral condylar overlap and/or shorter than 10 cm proximal tibial length. MRIs were not accessible in five of the patients. The remaining 78 patients were included in the study as Group A (multiple‐revision ACLR). To minimize bias, a control cohort (Group B) of 78 unilateral primary ACLR patients was selected in a 1:1 ratio corresponding to the 78 patients with multiple revisions (Group A). Direct matching by age or graft type was not feasible because only the age at the latest revision and the final graft configuration were available for the multiple‐revision cohort, which often involved more than one graft type across prior procedures. Instead, chronological matching was performed to align the control cohort with the evolution of surgical techniques and graft usage trends over the study period. Specifically, two representative time frames (2013 onward and 2018 onward) were used to mirror the temporal distribution of Group A cases and to account for changes in graft preference and technical approach observed over the past decade. This approach ensured that both groups were exposed to comparable surgical environments and rehabilitation protocols corresponding to their respective eras. Given the limited matching variables and sample size, formal propensity‐score matching was not applied, but the chronological design minimized confounding related to era‐dependent surgical trends. The choice of a ≥ 2‐year follow‐up threshold for defining successful primary ACLR is supported by registry and cohort data indicating that most graft failures and revisions occur within the first 1–2 postoperative years [25]. No failure was defined as the absence of clinical instability, absence of symptoms suggestive of graft insufficiency and no radiological evidence of graft rupture.

**Figure 1:**
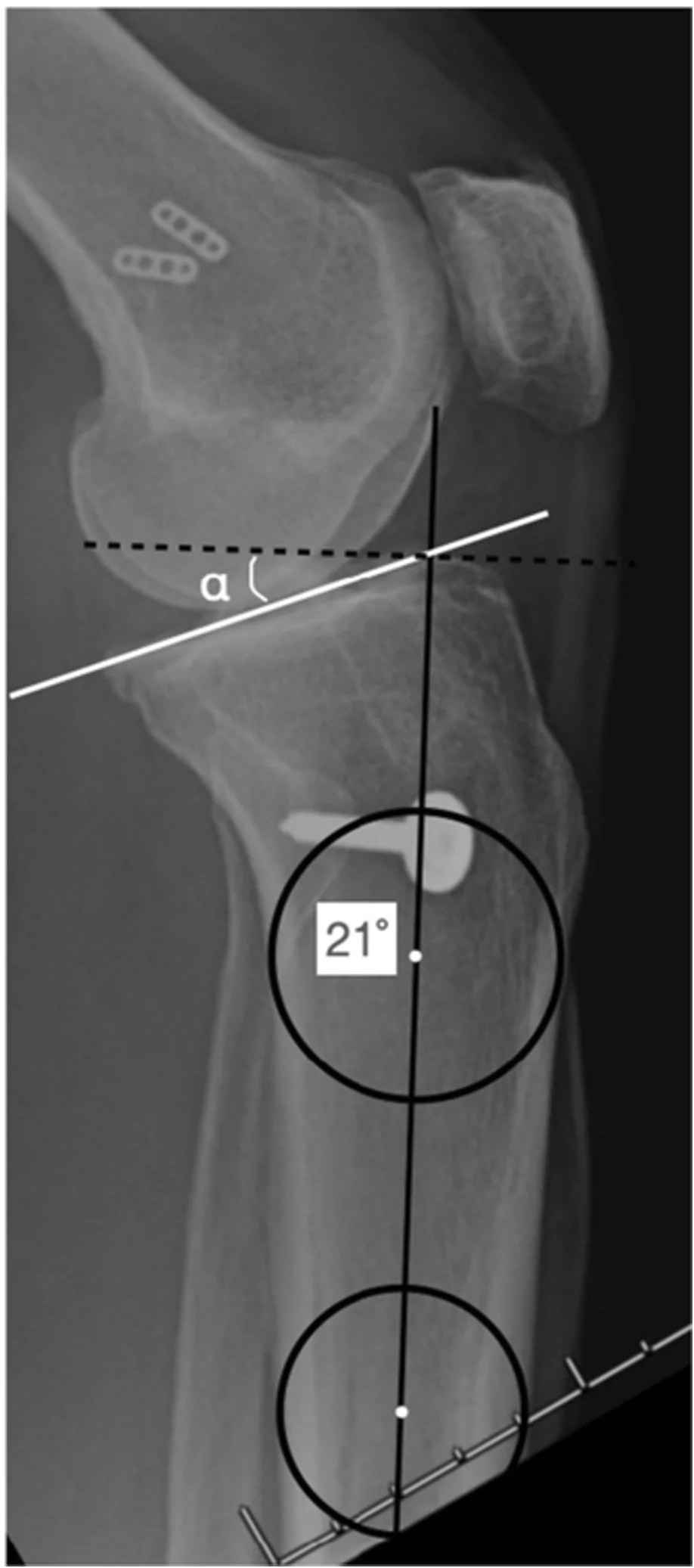
Posterior tibial slope (PTS) measurement on a true lateral knee radiograph using a standardized two‐circle method to define the tibial mechanical axis. Two circles were placed along the proximal tibial shaft: one right below the level of the tibial tuberosity and the other 5 cm distal to it. The solid line connecting their white dotted centres defines the proximal tibial axis. The angle *α* represents the PTS, calculated between the black dashed line perpendicular to this axis and a white line tangential to the subchondral surface of the medial tibial plateau. In this example, the PTS was measured as 21°.

Radiographic PTS was determined by measuring the angle between the medial tibial plateau and a line tangent to the proximal anatomic axis of the tibia. The proximal anatomic axis was defined using two circles placed along the tibial diaphysis, 5 and 10 cm below the tibial plateau (Figure 1).

**Figure 2:**
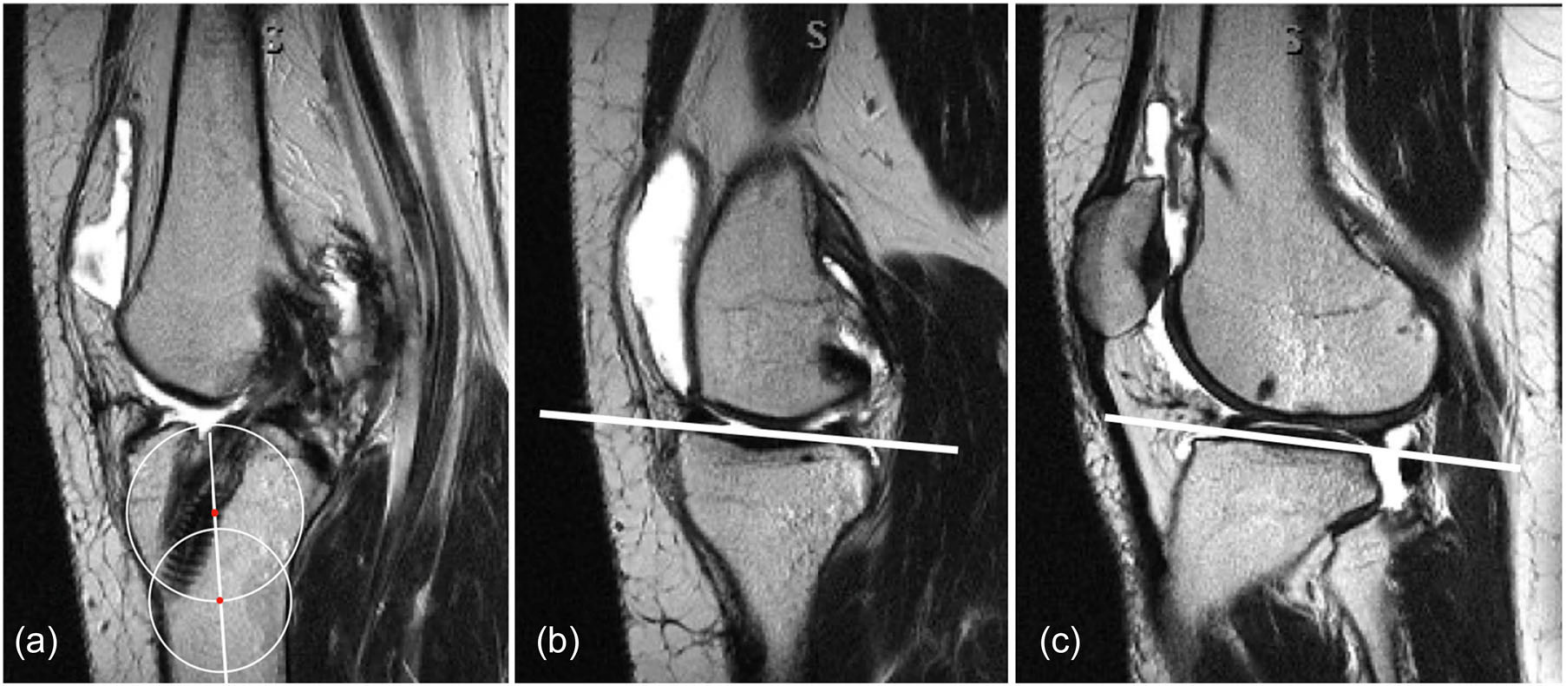
Measurement of medial and lateral posterior tibial slope (PTS) on sagittal knee magnetic resonance imaging (MRI). (a) Determination of the tibial longitudinal axis on the central sagittal slice using two best‐fit white circles placed within the proximal tibia. The cranial circle is positioned to touch the anterior, posterior and cranial cortices, while the caudal circle touches the anterior and posterior cortices, with its centre located on the circumference of the cranial circle. The centres of the circles are marked with red dots, and the tibial axis is defined by the white line connecting these two centres. (b) Measurement of the medial PTS on the slice showing the mediolateral centre of the medial plateau. A white tangent is drawn along the uppermost anterior and posterior cortices of the medial plateau, and the PTS is defined as the angle between this tangent and a line perpendicular to the tibial axis. (c) Measurement of the lateral PTS on the corresponding slice through the centre of the lateral plateau, using the same technique as in (b).

The commonly accepted PTS cutoff of 12° was derived from measurements taken 5 and 15 cm distal to the tibial joint line [21]. Research on the effect of proximal tibial length on defining the anatomic axis showed that PTS values were approximately 1° higher when the axis was set at 10 cm compared to 16 cm [7]. Therefore, a high‐risk PTS cutoff of ≥13° was used for further analysis, adjusting for shorter tibial length (10 cm) on measurements. Medial and lateral PTS were measured on MRI as defined previously [10] (Figure 2).

Minimal detectable change (MDC) with a 95% confidence interval (CI) of radiographic PTS was calculated with the measurements of 76 lateral knee radiographs by three independent raters in our previous study [15]. The mean MDC across the three raters was 1.0° (95% CI: 0.9°–1.1°). The reliability of PTS measurements was evaluated by two blinded raters.

### Statistical analysis

All statistical analyses were performed using SPSS Statistics (Version 25.0; IBM Corp.). The normality of continuous variables was assessed using the Shapiro–Wilk test. Continuous variables are reported as mean ± standard deviation (SD), whereas categorical variables are expressed as counts and percentages. Between‐group comparisons of continuous variables were conducted using independent‐samples *t* tests when normality assumptions were met, with Welch's correction applied when variances were unequal, and Mann–Whitney *U* tests when the data were not normally distributed. Categorical variables, including the proportions of patients exceeding predefined high‐risk PTS thresholds, were analysed using chi‐square tests or Fisher's exact tests when appropriate.

Associations between radiographic and MRI‐based PTS measurements were evaluated using Pearson's correlation coefficients for normally distributed variables and Spearman's rank correlation otherwise. Receiver operating characteristic (ROC) analysis was performed to determine the optimal radiographic PTS cutoff based on Youden's index, and the area under the curve (AUC) with its 95% CI quantified discriminatory ability. Logistic regression models were used to investigate the association between radiographic medial PTS and the likelihood of multiple revision ACLR. First, univariate logistic regression was performed, followed by a multivariable model adjusted for body mass index (BMI), sex, side, height and weight. Odds ratios (ORs) with 95% CIs were reported for each 1° increase in radiographic PTS. Reliability of radiographic and MRI‐based measurements was evaluated using Cronbach's *α* for both interobserver and intraobserver agreement, with *α* ≥ 0.80 indicating excellent reliability. A two‐sided *p* value < 0.05 was considered statistically significant.

## RESULTS

There were no significant demographic differences between patients undergoing primary ACLR and those with multiple‐revision ACLR. Mean weight, height and BMI were comparable between groups, and the sex distribution did not differ significantly (*p* = n.s.) (Table 1).

**Table 1:** Demographics and compared parameters between the groups.

Parameter	Primary ACLR group	Multiple‐revision ACLR group	*p* value
Sex (M/F)	36/42	41/37	n.s.
Weight (kg)	80 ± 18.1	82.5 ± 17.8	n.s.
Height (cm)	172.3 ± 10.9	173.2 ± 10	n.s.
BMI (kg/m^2^)	26.9 ± 5.3	27.4 ± 4.8	n.s.

Abbreviations: ACLR, anterior cruciate ligament reconstruction; BMI, body mass index; n.s., not significant.

Radiographic medial PTS was significantly higher in the multiple‐revision group compared with the primary ACLR cohort (12.5 ± 3.5° vs. 11.2 ± 3.0°, mean difference 1.3°, *p* = 0.020). The difference was higher than the reported MDC of 1.0°. In contrast, MRI‐based medial PTS, lateral PTS and medial–lateral PTS asymmetry did not differ significantly between groups (*p* = n.s.) for all comparisons. Mean values for radiographic PTS, MRI‐based medial and lateral PTS and PTS asymmetry values are represented in Table 2.

**Table 2:** Comparison of radiographic and MRI‐based posterior tibial PTS parameters between primary and multiple‐revision ACLR groups.

Measure	Primary ACLR group (min, max)	Multiple‐revision ACLR group (min, max)	*p* value
Radiographic medial PTS (°)	11.2 ± 3 (5, 17)	12.5 ± 3.5 (7, 22)	0.020
MRI medial PTS (°)	3.1 ± 2.8 (−4, 10)	3.5 ± 4.0 (−6, 13)	n.s.
MRI lateral PTS (°)	6 ± 3.4 (−3, 12)	6.5 ± 3.9 (−3, 16)	n.s.
PTS asymmetry (medial–lateral, MRI)	2.8 ± 3.4 (−5, 10)	3.1 ± 2.9 (−4, 11)	n.s.

Abbreviations: ACLR, anterior cruciate ligament reconstruction; MRI, magnetic resonance imaging; n.s., not significant; PTS, posterior tibial slope.

ROC analysis of radiographic medial PTS demonstrated an AUC of 0.58, reflecting modest discriminatory ability. The optimal threshold determined by Youden's index was 13°, corresponding to a sensitivity of 0.49 and specificity of 0.65. At this cutoff, the relative risk (RR) of multiple revisions was 1.41 (95% CI: 0.96–2.06) and the OR was 1.79 (95% CI: 0.94–3.42), though statistical significance was not achieved (*p* = n.s.). Similarly, thresholds of 14° and 15° were not significant predictors. In contrast, a radiographic medial PTS ≥ 16° was significantly associated with the risk of multiple‐revision ACLR, with an RR of 2.67 (95% CI: 1.10–6.46) and an OR of 3.10 (95% CI: 1.14–8.40), *p* = 0.040. At this threshold, specificity improved markedly to 0.91, and the positive predictive value was 0.70, indicating that a PTS ≥ 16° reliably identifies high‐risk individuals (Table 3).

**Table 3:** Association of radiographic PTS thresholds with multiple‐revision ACL reconstruction risk.

Threshold (°)	Primary ACLR group: *n*/*N* (%)	Multiple‐revision ACLR group: *n*/*N* (%)	RR [95% CI]	OR [95% CI]	Fisher's exact *p*
≥13°	27/78 (34.6%)	38/78 (48.7%)	1.41 [0.962, 2.059]	1.79 [0.942, 3.417]	n.s.
≥14°	18/78 (23.1%)	27/78 (34.6%)	1.50 [0.903, 2.491]	1.76 [0.873, 3.567]	n.s.
≥15°	14/78 (17.9%)	19/78 (24.4%)	1.36 [0.734, 2.510]	1.47 [0.678, 3.198]	n.s.
≥16°	6/78 (7.7%)	16/78 (20.5%)	2.67 [1.101, 6.456]	3.10 [1.142, 8.400]	0.040

Abbreviations: ACLR, anterior cruciate ligament reconstruction; CI, confidence interval indicates the 95% range of plausible true effect sizes; n.s., not significant; OR, odds ratio expresses the ratio of odds of multiple revisions above versus below the threshold; PTS, posterior tibial slope; RR, relative risk represents the probability of multiple revisions in patients above each PTS threshold compared to those below it.

Univariate logistic regression revealed that each 1° increase in radiographic medial PTS was associated with a 10% increase in the odds of multiple revision ACLR (OR = 1.10, 95% CI: 1.00–1.22, *p* = 0.049). After adjusting for BMI, sex, side, height and weight, this association remained statistically significant, with an adjusted OR of 1.11 (95% CI: 1.01–1.23, *p* = 0.034).

**Figure 3:**
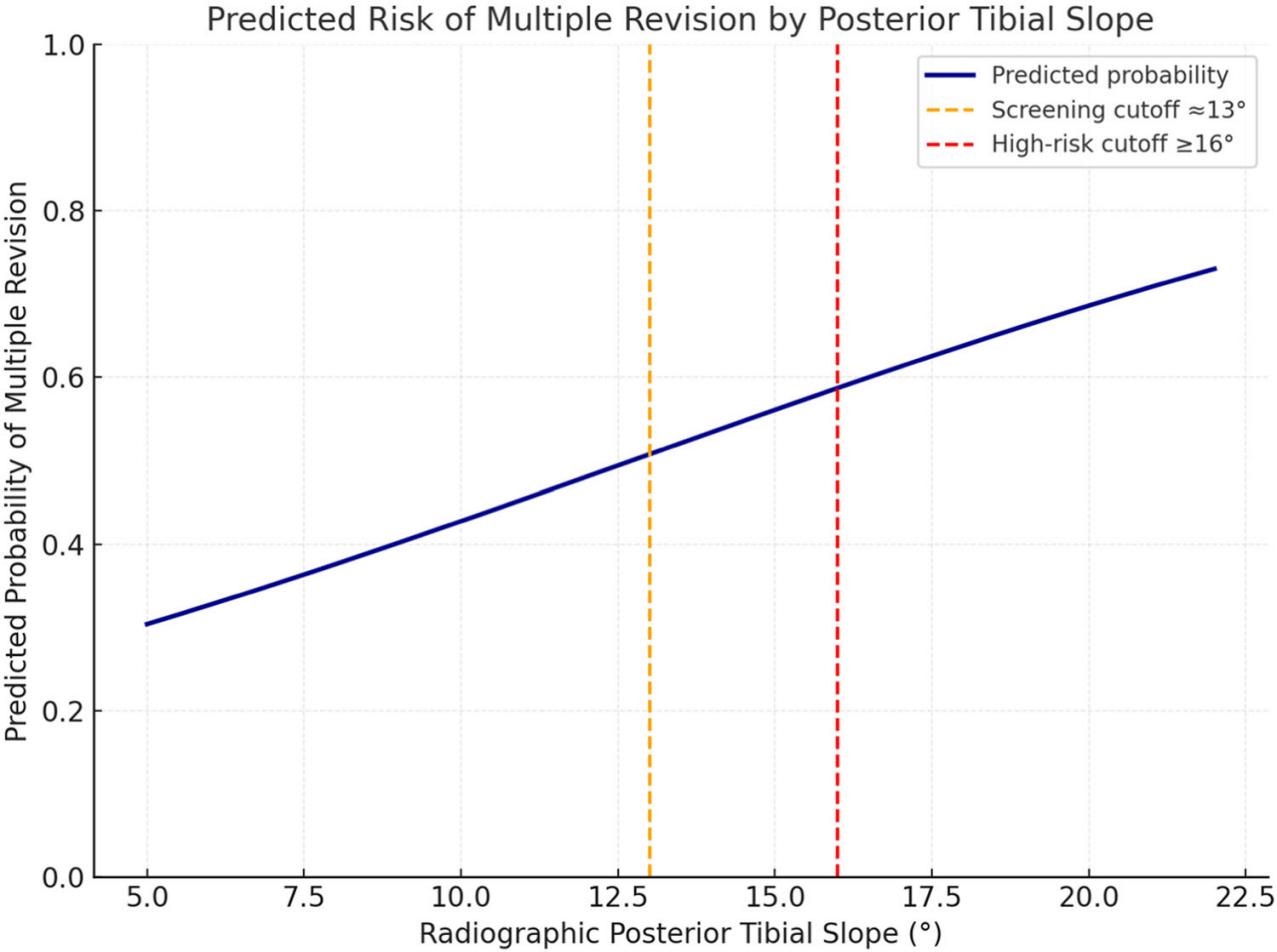
Predicted probability of multiple‐revision anterior cruciate ligament reconstruction as a function of radiographic medial posterior tibial slope, based on the multivariable logistic regression model. The blue line represents the predicted probability curve, the yellow dashed line at 13° marks the screening cutoff, and the red dashed line at 16° indicates the high‐risk threshold.

Figure 3 illustrates the predicted probability of belonging to the multiple‐revision ACLR group as a function of radiographic medial PTS, based on the adjusted multivariable logistic regression model. The model demonstrates a progressive, nonlinear increase in predicted risk with higher PTS values. A clinically relevant three‐zone risk stratification emerges from these findings. Patients with PTS < 13° represent a low‐risk zone, those with PTS between 13° and 16° constitute an intermediate‐risk zone, and patients with PTS ≥ 16° fall into a high‐risk zone. At 13°, the adjusted predicted probability of multiple‐revision ACLR is approximately 50.7%, with an RR of 1.41 (95% CI, 0.96–2.06) compared with PTSs <13°. At 16°, the predicted probability increases to approximately 58.7%, corresponding to a significantly elevated risk (RR = 2.29, 95% CI: 1.00–5.25) with excellent specificity (0.91) and a positive predictive value of 0.70.

Correlation analyses demonstrated a moderate association between radiographic PTS and medial PTS on MRI in both groups (*r* = 0.49, *p* < 0.001 for the primary ACLR cohort; *r* = 0.43, *p* < 0.001 for the multiple‐revision cohort). Lateral PTS on MRI showed no significant correlation with radiographic PTS in the multiple‐revision group but demonstrated a weak association in the primary group (*r* = 0.29, *p* = 0.01). These results should be interpreted in light of the differing tibial shaft lengths visualized on MRI versus radiographs, which may influence PTS measurement alignment and reduce cross‐modality agreement.

Interobserver reliability was excellent (Cronbach's *α* = 0.902 and 0.851), as was intraobserver reliability for both radiographic and MRI measurements (Cronbach's *α* = 0.924 and 0.852), respectively.

## DISCUSSION

This study demonstrates that radiographic medial PTS is a reliable anatomical parameter distinguishing patients who underwent multiple revision ACLR from those with successful primary ACLR. A higher radiographic medial PTS was consistently associated with increased revision susceptibility, underscoring its relevance for clinical risk assessment. In contrast, MRI‐derived medial and lateral PTS measurements, as well as medial–lateral PTS differences, did not contribute meaningful or incremental predictive value. Overall, these findings emphasize the practical utility of radiographic PTS assessment and the clinical importance of even modest variations in tibial posterior slope when considering revision risk.

The current study findings are consistent with prior studies suggesting that an increased PTS predisposes to ACL graft failure [1, 6, 16], but several novel insights are presented. Unlike most previous studies, this study directly compared radiographic and MRI‐derived medial and lateral PTS within the same cohort. The results demonstrate that radiographic medial PTS better differentiates high‐risk patients, whereas MRI‐derived PTSs, despite showing moderate correlation with radiographic values, failed to discriminate between patients with successful primary ACLR and those undergoing multiple revisions. These discrepancies likely reflect differences in imaging planes, reference axes, tibial length and measurement reproducibility across modalities.

Previous studies identified variable cutoff values for PTS in terms of failure prediction. For example, a global PTS ≥ 17° measured on whole‐leg radiographs was associated with an OR of 15.6 for graft failure, while another study reported that PTS ≥ 16°, when addressed with PTS‐reducing osteotomy, significantly lowered graft failure rates and improved knee stability compared to isolated ACLR [20, 27]. Conversely, Dæhlin et al. found no association between global PTS on full‐leg radiographs and revision risk [3]. Importantly, using an anatomic tibial axis yields medial PTS values comparable to those measured with a 15‐cm proximal tibial axis, supporting the reliability of standardized radiographic measurement techniques. A clinical study further demonstrated that patients with excessive PTS (average 18.5°) treated with PTS‐reducing osteotomy experienced improved stability and no failures at 2 years [22].

Our analysis refines the understanding of PTS thresholds by showing that while 13° marks the transition to elevated risk, only ≥16° reliably identifies a high‐risk subgroup with clinically meaningful predictive accuracy. This finding has direct implications for preoperative planning and patient counselling. The identified ≥16° threshold should not be viewed as a substitute for the established 12° cutoff related to primary ACL injury risk, but rather as an indicator of markedly increased risk for recurrent graft failure among previously reconstructed knees.

These findings underscore the value of radiographic medial PTS as a practical, widely available and cost‐effective screening tool for risk stratification in ACLR. Identifying patients with a PTS of ≥16° preoperatively allows for improved shared decision‐making, better counselling regarding revision risk and consideration of PTS‐reducing osteotomies in select high‐risk cases. In contrast, MRI‐derived medial and lateral PTSs provide no additional predictive benefit beyond radiographs, suggesting their limited utility in routine risk assessment.

The modest discriminatory ability of radiographic medial PTS (AUC = 0.58) highlights that while PTS is an independent predictor, it explains only part of the variance in multiple‐revision risk. Other factors such as tunnel placement, graft size, generalized laxity and rehabilitation protocols likely interact with PTS to influence outcomes [28]. Taken together, 13° serves as a reasonable screening threshold for risk stratification, while ≥16° reliably identifies a subgroup at significantly elevated risk for multiple‐revision ACLR. Our findings align with a recent opinion, where PTS‐reducing osteotomies are proposed for select patients in primary ACLR [5].

Limitations include the retrospective design and single‐centre setting, which may limit generalizability. Important surgical variables such as tunnel placement, graft size and concomitant meniscal pathology were not available for all patients and, therefore, could not be incorporated into the analysis; these factors are known contributors to ACLR outcomes and represent potential confounders when interpreting associations between PTS and revision risk. Additionally, static anterior tibial translation (SATT) was not assessed, despite its relevance in characterizing sagittal tibial position and its potential interaction with PTS‐related biomechanics; the absence of uniformly available SATT measurements prevented its inclusion. Finally, although alternative MRI‐based slope assessment techniques may yield different results, our standardized measurement protocol enhances reproducibility.

## CONCLUSION

Radiographic medial PTS is the independent predictor of multiple revision ACLR, whereas MRI‐derived medial and lateral PTSs provide no additional discriminatory value. A radiographic medial PTS of ≥16° measured using a 10‐cm proximal tibial axis was associated with an approximately threefold increase in the odds of multiple revisions. These findings support prioritizing radiographic medial PTS in preoperative risk stratification and indicate limited clinical utility for MRI‐derived medial or lateral PTS measurements, as well as medial–lateral PTS differences, in surgical decision‐making.

## AUTHOR CONTRIBUTIONS

All listed authors have contributed substantially to this work: All authors have read and approved the final manuscript to be submitted and published.

## CONFLICT OF INTEREST STATEMENT

Mahmut Enes Kayaalp is a Deputy Editor‐in‐Chief of Knee Surgery, Sports Traumatology, Arthroscopy (KSSTA), Co‐editor of Joint Diseases and Related Surgery and Member of the ESSKA U‐45 Scientific Committee. Volker Musahl reports educational grants, consulting fees and speaking fees from Smith & Nephew, educational grants from Arthrex and DePuy/Synthes and is a board member of the International Society of Arthroscopy, Knee Surgery and Orthopaedic Sports Medicine (ISAKOS) and deputy editor‐in‐chief of Knee Surgery, Sports Traumatology, Arthroscopy. The remaining authors declare no conflict of interest.

## ETHICS STATEMENT

This study received approval from IRB:STUDY19030196 University of Pittsburgh.

## Data Availability

Data are available upon request.
